# Biologic therapy for refractory pemphigoid gestationis: An evidence-based systematic review

**DOI:** 10.1016/j.jdin.2023.08.015

**Published:** 2023-08-31

**Authors:** Siddhartha Sood, Jensen Yeung, Asfandyar Mufti

**Affiliations:** a Temerty Faculty of Medicine, University of Toronto, Toronto, Ontario, Canada; b Division of Dermatology, Department of Medicine, University of Toronto, Toronto, Ontario, Canada; c Department of Dermatology, Sunnybrook Health Sciences Centre, Toronto, Ontario, Canada; d Department of Dermatology, Women's College Hospital, Toronto, Ontario, Canada; e Probity Medical Research, Waterloo, Ontario, Canada

**Keywords:** biologics, evidence-based, immunobullous, pemphigoid gestationis, pregnancy

*To the Editor:* Pemphigoid gestationis (PG) is a rare inflammatory condition of pregnancy characterized by vesicobullous eruption and severe pruritus.^1^ It has been linked to maternal and fetal complications.^1^ Although use of biologics for refractory PG remains novel, this systematic review summarizes evidence surrounding their use for this condition.

Following Preferred Reporting Items for Systematic Reviews and Meta-Analyses guidelines, we searched Embase and MEDLINE databases using specific keywords (Supplementary Table I, available via Mendeley at https://doi.org/10.17632/hxhtnx5g7r.1). Quality of evidence was assessed using Oxford Centre for Evidence-Based Medicine 2011 Levels of Evidence. After independent screening by 2 reviewers, 20 articles (publication date: 1998-2023) reflecting 25 patients and 28 treatments were included (Fig 1; Supplemental Table II, available via Mendeley at https://doi.org/10.17632/hxhtnx5g7r.1). The mean age of mothers was 33.1 years (range: 17-44 years). The mean time-to-onset of patients with PG in the antepartum period was 5.2 months (24/25). PG continued into the postpartum period for 60% (15/25) of cases. There were PG episodes during previous pregnancies in 6 (24%) instances. All patients previously failed nonbiologic systemic therapy including systemic corticosteroids (100%) (Supplementary Table II).

**Fig 1:**
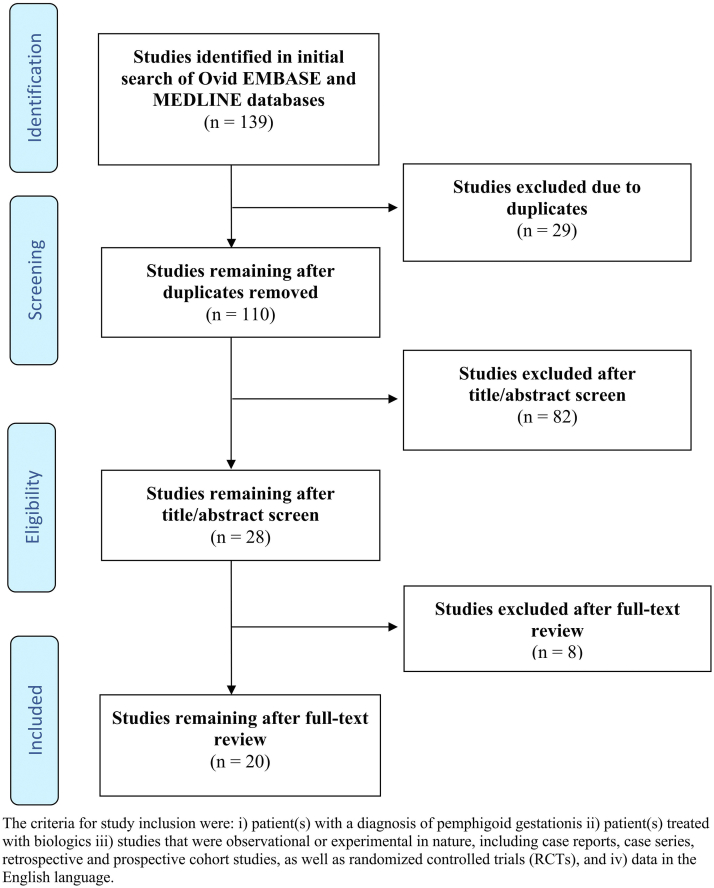
Flow diagram of literature screening using the Preferred Reporting Items for Systematic Reviews and Meta-Analyses guidelines.

Figure adapted from http://prisma-statement.org.

The mean treatment duration was 94.2 days (25/28). All patients used systemic concomitant medications of which prednisone/prednisolone was most common (92%, 23/25) (Supplementary Table II). Intravenous immunoglobulin (IVIg) (66%, 18/27) was the most frequently used monotherapy for PG, followed by omalizumab (14.8%, 4/27), dupilumab (11.1%, 3/27), and rituximab (7.4%, 2/27). There was 1 (3.6%) instance of combination treatment with IVIg/rituximab. Complete resolution was observed with dupilumab (100%, 3/3), rituximab (100%, 2/2), IVIg/rituximab combination (100%, 1/1), IVIg (72.2%, 13/18), and omalizumab (50%, 2/4) (Supplementary Table III, available via Mendeley at https://doi.org/10.17632/hxhtnx5g7r.1). In 2 (11.1%) reported cases, a reduction of 95.5% was documented in Bullous Pemphigoid Disease Area Index with IVIg use (Supplementary Table III). Six (21.4%) patients experienced recurrence following initial resolution of PG (Supplementary Table II). Fetal complications occurred in 6 (21.4%) patients, including preterm premature rupture of membranes (33.3%, 2/6). Maternal adverse events occurred in 1 (3.5%) patient receiving rituximab, reflecting peripheral leukocytosis and raised liver function tests; neither led to discontinuation (Supplementary Table II).

Mechanistically, the pathogenesis of PG may be analogous to bullous pemphigoid (BP), with evidence for the latter suggesting Th2-predominant immune dysregulation alongside anti-BP180 autoantibody formation.^1^^,^^2^ These findings may explain the favorable outcomes observed with cytokine inhibition through dupilumab as well as antibody-level targeting through IVIg, omalizumab, and rituximab. Overall, these agents may decrease serum anti-BP180 which has been linked to adverse pregnancy outcomes in PG.^3^ Currently, first-line treatment for PG involves systemic corticosteroids with a response rate of 79.7%; however, a recurrence rate of 65.8% suggests additional therapy may be necessary.^4^ Our results with biologics for PG refractory to corticosteroids are comparable to this response rate. While no internationally accepted guidelines exist for PG, recent BP treatment guidelines have recommended the above agents as third-line therapy.^5^ Of these biologics, IVIg has an established safety profile permissible during pregnancy and breastfeeding.^1^ We observed limited fetal complications in this review.

Study limitations include small sample size, concomitant medication use, and potential publication bias. Regardless, we highlight evidence on the utility of biologics for refractory PG. Larger-scale studies are warranted.

## Conflicts of interest

Dr Yeung has been an advisor, consultant, speaker, and/or investigator for AbbVie, Allergan, Amgen, Astellas, Bausche, Baxalta, Boehringer Ingelheim, Celgene, Centocor, Coherus, Dermira, Eli Lilly, Forward, Fresnius Kabi, Galderma, Incyte, Janssen, LEO Pharma, Lilly, Medimmune, Merck, Novartis, Pfizer, Regeneron, Roche, Sanofi Genzyme, Sun Pharma, Takeda, UCB, and Xenon. Dr Mufti has been a speaker for AbbVie and Janssen. Author Sood has no conflicts of interest to declare.
